# The Association of Problematic Online Gaming Behavior With Mental Well-Being and Depressive Symptoms Among Students of Professional Colleges in Rishikesh

**DOI:** 10.7759/cureus.22007

**Published:** 2022-02-08

**Authors:** Rishita Chandra, Santosh Kumar, Yogesh Bahurupi, Vikram Singh Rawat

**Affiliations:** 1 Public Health, All India Institute of Medical Sciences, Rishikesh, Rishikesh, IND; 2 Family and Community Medicine, All India Institute of Medical Sciences, Rishikesh, Rishikesh, IND; 3 Psychiatry, All India Institute of Medical Sciences, Rishikesh, Rishikesh, IND

**Keywords:** internet gaming disorder, depression, well-being, problematic online gaming, problematic online gaming behaviour

## Abstract

Background and objective

Excessive online gaming is becoming a behavior pattern, and it has been recently classified as a medical illness and added to the International Classification of Diseases-11 (ICD-11), termed as "gaming disorder". This condition can be severe enough to result in significant impairment in psychological health. In light of this, we conducted this study to analyze the relationship between problematic online gaming and mental well-being among students of professional colleges in Rishikesh.

Methods

A cross-sectional study was conducted among professional college students in Rishikesh on problematic online gaming behavior (POGB). The Problematic Online Gaming Questionnaire-Short Form (POGQ-SF) was used to assess POGB among the participants. The five-item World Health Organization Well-Being Index (WHO-5) and Patient Health Questionnaire-9 (PHQ-9) were used to assess mental well-being and depressive symptoms respectively.

Results

The prevalence of POGB was 27.4% among our cohort, and its association with mental well-being as well as depressive symptoms was statistically significant. The study participants with poor mental well-being and mild or moderate to moderately severe depressive symptoms had higher odds of developing POGB.

Conclusion

POGB has been recently identified as a behavioral addiction and it is often neglected. A significant proportion of the study participants exhibited problematic gaming behavior.

## Introduction

The urge to engage in some form of recreational activity is an essential element of human biology and psychology. The things that individuals do off the job, such as leisure activities, and the lifestyles they lead are caught up in various forces of change and influence their health and psychological well-being. Online video games are a popular recreational activity among many individuals worldwide [1]. Levitis et al. have defined the term behavior as "internally coordinated responses (actions or inactions) of whole living organisms (individuals or groups) to internal and or external stimuli, excluding responses more easily understood as developmental changes" [2].

A state of craving or urge has been reported by many people suffering from behavioral addiction before the initiation of that behavior, which simulates the pattern, and it has been observed among individuals with substance use disorders prior to the start of their addiction. These types of behaviors are found to provide a state of "high" or positive, light, and cheerful mood, similar to the one reported in substance abuse. The cause of both behavioral and substance use disorders may be attributed to the dysregulation of emotions [3]. The incidents of individuals suffering from "non-drug addictions or behavioral addictions" have been reportedly on the rise, and many such individuals are approaching clinics with complaints of compulsive habits, e.g., eating, exercising, gambling, shopping, and playing video games [4].

As video gaming has gained popularity in many parts of the world, it has become a matter of concern among students. Excessive gaming is becoming a behavior pattern that can be of sufficient severity to result in significant impairment in personal, family, social, educational, occupational, and other important areas of functioning [5]. Gaming disorder, both online and offline variants, has recently been added to the International Classification of Diseases-11 (ICD-11) as a clinically recognizable and significant syndrome. The World Health Organization (WHO) states that "when the pattern of gaming behavior is of such a nature and intensity, it results in marked distress or significant impairment in personal, family, social, educational, or occupational functioning" [6].

The features of online video gaming enable players to communicate with fellow gamers in real-time. They not only cooperate during the game but also engage in competition at will, which then leads to the phenomenon of immediate social comparison. The structural characteristics inbuilt in these games provide no fixed endpoint, and the gamers continue to play with the curiosity related to the challenges and rewards lying ahead in further stages. The virtual world of online gaming provides a platform to the gamers and instills a feeling of confidence and provides a niche where they can connect easily to escape a reality that they find dissatisfying. Cheap internet connections have provided easy access to gadgets and the internet has become one of the causes for promoting non-drug addictions such as online gaming and gambling. The "gamers" often compulsively indulge in gaming, neglecting other interests, and their persistent and recurrent online engagement results in clinically significant impairment or distress. The activity of gaming triggers a neurological response that influences feelings of pleasure and reward, and the result, in its extreme form, is manifested as addictive behavior [7].

Problematic gaming behavior may be associated with various characteristics and consequences related to psychological and social health. Video game addicts can also suffer from cognitive impairments [8]. Psychological morbidities such as depression, anxiety, fatigue, disturbed sleep, trouble in concentration, and low satisfaction with life are related to problematic gaming behavior. The predictors of problematic gaming can be depression, a low level of physical activity, and a marked increase in online interaction. All these psychosocial health issues play a pivotal role among problematic gamers [9].

In Goa, India, the prevalence of problematic online gaming behavior (POGB) has been reported to be 8% [10]. Another study conducted among the students at a medical college has found that students who indulged in addictive gaming reported psychosocial disturbances like mood disorders, depression, disturbed sleep, and anxiety [5]. A cross-sectional study conducted in Guntur, Andhra Pradesh has reported that 12.3% of participants were using the internet for online gaming. Mobile phones were used by about 63% of students to access the internet [11].

According to Wartberg et al., the psychosocial and behavioral makeups of online gamers are associated with a wide range of negative consequences. The level of social support they receive has decreased, and their health-related quality of life has deteriorated. They frequently show social phobia, problems in controlling anger, emotional distress, and decreased self-esteem. Hence, identifying and analyzing these correlates is of vital significance in designing and implementing intervention programs for problematic online gaming [12].

The present study aimed to estimate the prevalence of POGB and its psychological correlates among professional college students in Rishikesh. Identification of such groups will help to assess psychological morbidities associated with addictive online gaming and correlate its impact with their mental health and well-being.

## Materials and methods

Study design and setting

This cross-sectional study was conducted among students at professional colleges, both public and private, in Rishikesh. Due to the ongoing coronavirus disease 2019 (COVID-19) pandemic, telephonic and online methods of data collection via Google Forms were used over a period of two months, from August 2020 to September 2020. A multistage random sampling method was used to recruit participants for the study. In the first stage, four out of eight professional colleges were selected using simple random sampling followed by a selection of a few departments/courses from the institutes. In the third stage, students fulfilling the eligibility criteria were recruited for the study.

Study participants

A total of 453 students aged 18 to 24 years who fulfilled the eligibility criteria and provided informed consent were recruited for the study from professional colleges. Students of both genders who used electronic gadgets and the internet were included. All study participants were either involved in online gaming during the study period or had been involved sometime in the past.

Sample size

The prevalence of POGB as reported in a previous study done by Bicholkar et al. in 2019 in Goa, India is 8%. Based on this data, the sample size for the current study was calculated using the following formulae:

Sample size, n = [DEFF*Np (1-p)]/[(d2/Z 21-α/2 *(N-1)+p*(1-p)]

Population size (for finite population correction factor or FPC) (N) = 6,960

Hypothesized % frequency of outcome factor in the population (p) = 8%

Confidence limits as % of 100 (absolute +/- %) (d) = 2.5%

Design effect = 1

Confidence level (%) = 95%

Sample size = 425

The estimated sample size = 425

In this study, responses from 453 participants were received.

Procedure

The students were given a telephonic briefing session about the study, and their concerns and doubts prior to sharing the self-administered questionnaire via Google Forms on social media platforms such as WhatsApp and Gmail were addressed.

Study tool and variables

The study tool included a semi-structured, pretested, and validated questionnaire detailing sociodemographic patterns and general information along with pretested scales. A self-administered questionnaire based on the 12-item version of the Problematic Online Gaming Questionnaire (POGQ-SF) [13] was used to assess problematic online gaming among students. Additionally, psychological characteristics were assessed using the five-item World Health Organization Well-Being Index (WHO-5) questionnaire [14], which is a short five-item instrument designed to measure current mental well-being. The Patient Health Questionnaire-9 (PHQ-9) [15] was used to screen for symptoms of depression over the past two weeks.

The study variables included the presence and absence of POGB, sociodemographic information (e.g., age, sex, education and occupation of the head of the family, family type, number of family members, and monthly income of the family head). The personal details of the study participants included residential status, academic status, and relationship status. The components of gaming behavior included the duration of playing games, mode and gaming format mostly used, the device used, the type of games the students liked the most, and the preferred time of the day for playing games.

Data entry and analysis

The data from the Google Forms were exported to Microsoft Excel 2013 and then to SPSS Statistics version 23 (IBM, Armonk, NY) for analysis. Descriptive statistics were used to describe categorical variables as proportions or percentages. In inferential statistics, the chi-squared test was used to find the association of POGB with sociodemographic factors such as age, education, occupation, socioeconomic status, and components of gaming behavior such as the duration of playing games, mode and format of gaming, devices used, and the preferred time of the day for playing play games. The strength of the association of POGB with mental well-being and depressive symptoms was calculated by applying multinomial logistic regression to determine the odds ratio (OR). Multivariate logistic regression was applied to minimize the effects of confounding factors and to predict the associated risk factors.

## Results

A total of 453 participants were recruited for the study. The mean age of the participants was 21.51 ±1.72 years, and all of them belonged to the age group of 18-24 years. The majority of the study participants were women (71.1%), while males accounted for 28.9% of the cohort. The majority belonged to the upper middle class, 60.7%, with 24.9% from the upper class. Of the total study participants, 14.3% belonged to the lower middle class, and there were no study participants from the lower class. The distribution of study participants according to sociodemographic variables is presented in Table 1.

**Table 1:** Distribution of study participants according to sociodemographic variables (n = 453) *Modified Kuppuswamy Scale

Sociodemographic variables	Subcategories	Frequency (N)	Percentage (%)
Age group, years	18-21	211	46.6
	22-24	242	53.4
Sex	Male	131	28.9
	Female	322	71.1
Religion	Hindu	427	94.3
	Muslim	19	4.2
	Sikh/Christian	7	1.50
Family type	Nuclear	329	72.6
	Joint	109	24.1
	Single parent/extended family	15	3.3
Educational qualification of the head of the family*	Professional	56	12.4
	Graduate	301	66.4
	Intermediate/diploma	69	15.2
	High school	27	6.0
Occupation of the head of the family*	Professional	204	45.0
	Semi-professional	131	28.9
	Clerk/shop/farm	66	14.6
	Skilled	31	6.8
	Semi-skilled	19	4.2
	Unskilled	2	0.4
Socioeconomic status*	Upper class	113	24.9
	Upper middle class	275	60.7
	Lower middle class	65	14.3
	Total	453	100.0

The majority of the participants were from dental science and paramedical courses (60%); 15.9% were from management and technology fields, followed by 16.6% from applied sciences. A few (7.5%) were from the commerce stream. Among the participants, the majority (70.2%) engaged in both online and offline gaming, while 29.8% used online gaming only. None of the participants used offline gaming mode alone. As for the distribution of the study participants based on their preference of the device for playing online games, the majority (81.5%) used mobile/tablet, while 10.6% used PC/laptop, and 7.5% used consoles such as PlayStation™, Xbox, etc. Very few (0.4%) used all of the above-mentioned gaming devices. Regarding the most preferred gaming format, 51.4% of study participants used the multi-player gaming format, followed by 41.3% using the single-player gaming format. A few (7.3%) of the study participants used a massively multiplayer gaming format. About 47.2% of study participants liked playing games before going to sleep, followed by 43.9% who liked to play after college hours; 7.3% liked playing games during recess hours and 1.5% liked playing games before college hours. The majority of students (45.7%) were involved in gaming for less than an hour, while 39.3% played for one to two hours, 13.5% for three to five hours, and 1.5% for more than five hours.

Problematic online gaming behavior, mental well-being, and symptoms of depression

As per the estimation of prevalence using POGQ-SF, POGB was observed in 27.4% (n = 124) of the study participants. Of the total 453 participants, 66.4% reported a normal state of well-being, followed by 20.8% with poor well-being, and 12.8% of the participants had issues indicative of depression as per the WHO-5 criteria. The estimation of the symptoms of depression using the PHQ-9 indicated that 21.2% (n = 96) had mild depression, followed by 5.1% (n = 23) with moderate to moderately severe depression. About 73.7% (n = 334) had no or minimal symptoms of depression.

Association of problematic online gaming behavior with mental well-being and depressive symptoms of the participants

The association of POGB with mental well-being (χ2 = 36.00, p = 0.0001) and depressive symptoms (χ2 = 49.711, p = 0.0001) among the study participants were found to be statistically significant. The multinomial regression suggested that participants with poor well-being had 3.102 times higher odds of having POGB as compared to those with a normal state of well-being, and participants who had issues indicative of depression had 4.375 times higher odds of having POGB (Table 2). The participants with mild symptoms of depression had 3.191 times higher odds of being affected by POGB as compared to participants with no or minimal symptoms; participants who were found to have moderate and moderately severe symptoms of depression had 2.901 times higher odds of suffering from POGB (Table 3).

**Table 2:** Problematic online gaming behavior and its association with the mental well-being of the respondents (n = 453) *Statistically significant. ^†^Multinomial regression was applied OR: odds ratio; CI: confidence interval

Problematic online gaming behavior	Total, n (%)	Mental well-being^†^			Chi-squared test
		Normal well-being, n (%)	Poor well-being, n (%)	Indicative of depression, n (%)	
Present	124 (27.4)	56 (18.6)	39 (41.5)	29 (50.0)	36.000, p-value = 0.0001*, Df = 2
Absent	329 (72.6)	245 (81.4)	55 (58.5)	29 (50.0)	
Total	453 (100)	301 (100)	94 (100)	58 (100)	
OR (95% CI)		1	3.102 (1.877-5.128)	4.375 (2.423-7.900)	

**Table 3:** Problematic online gaming behavior and its association with depressive symptoms of the respondents (n = 453) *Statistically significant. †Multinomial regression was applied OR: odds ratio; CI: confidence interval

Problematic online gaming behavior	Total, n (%)	Depressive symptoms^†^			Chi-squared test
		Minimal or none, n (%)	Mild, n (%)	Moderate + moderately severe, n (%)	
Present	124 (27.4)	70 (21.0)	44 (45.8)	10 (43.5)	49.711, p-value = 0.0001*, df = 5
Absent	329 (72.6)	264 (79.0)	52 (54.2)	13 (56.5)	
Total	453 (100)	334 (100)	96 (100)	23 (100)	
OR (95% CI)		1	3.191 (1.974-5.158)	2.901 (1.221-6.893)	

The association of problematic online gaming behavior with demographics and gaming variables

The association of POGB with demographic and gaming variables is shown in Table 4. Chi-squared analyses suggested that POGB was significantly associated with age [χ2 = 14.071, p = 0.0001, OR (95% CI) = 2.273 (1.472-3.509)], sex [χ2 = 11.738, p = 0.001, OR (95% CI) = 0.508 (0.328-0.788)], and place of residence [χ2 = 9.477, p = 0.002, OR (95% CI) = 1.919 (1.263-2.914)]. Also, POGB was found to be statistically significant with respect to other gaming variables such as duration of gaming [χ2 = 80.364, p = 0.001, OR (95% CI) = 10.660 (5.929-19.166)], mode of gaming [χ2 = 30.691, p = 0.001, OR (95% CI) = 0.300 (0.194-0.464)], gaming format [χ2 = 22.55, p = 0.001, OR (95% CI) = 0.330 (0.207-0.528)], and devices used for gaming [χ2 = 32.440, p = 0.001, OR (95% CI) = 3.974 (2.423-6.516)]. No significant association was found between preferred gaming time and POGB.

**Table 4:** Association of problematic online gaming behavior with demographics and gaming variables (n = 453) *Statistically significant OR: odds ratio; CI: confidence interval; PG: paying guest

Demographics and gaming variables	Total, n (%)	Problematic online gaming behavior		OR (95% CI)	Chi-squared test
		Present, n (%)	Absent, n (%)		
Age group, years					
18-21	211 (46.5)	40 (32.3)	171 (52.0)	2.273 (1.472-3.509)	14.071, p-value = 0.0001*
22-24	242 (53.5)	84 (67.7)	158 (48.0)		
Sex					
Male	131 (28.9)	49 (39.5)	82 (24.9)	0.508 (0.328-0.788)	11.738, p-value = 0.001*
Female	322 (71.1)	75 (60.5)	247 (75.1)		
Place of residence					
Home with family	268 (59.2)	59 (47.6)	209 (63.5)	1.919 (1.263-2.914)	9.477, p-value = 0.002*
Hostel/PG/tenants	185 (40.8)	65 (52.4)	120 (36.5)		
Duration of gaming per day					
≥3 hours	385 (85.0)	75 (60.5)	310 (94.2)	10.660 (5.929-19.166)	80.364, p-value = 0.0001*
<3 hours	68 (15.0)	49 (39.5)	19 (5.8)		
Devices preferred for gaming					
Mobile/tablet	369 (81.5)	80 (64.5)	289 (87.8)	3.974 (2.423-6.516)	32.440, p-value = 0.0001*
Other devices (PC/laptop/consoles)	84 (18.5)	44 (35.5)	40 (12.2)		
Mode of gaming					
Online	135 (29.8)	61 (49.2)	74 (22.5)	0.300 (0.194-0.464)	30.691, p-value = 0.0001*
Both online and offline	318 (70.2)	63 (50.8)	225 (77.5)		
Gaming format					
Multi-player	266 (58.7)	95 (76.6)	171 (52.0)	0.330 (0.207-0.528)	22.552, p-value = 0.0001*
Single-player	187 (41.3)	29 (23.4)	158 (48.0)		
Most preferred time for gaming					
Before going to sleep	214 (47.2)	59 (46.6)	155 (47.1)	0.981 (0.649-1.484)	0.008, p-value = 0.929
Other times during the day (before college/recess/evening)	239 (52.8)	65 (52.4)	174 (52.9)		

Logistic regression analysis was applied to minimize the confounding effect of risk factors for POGB among students at professional colleges. The Hosmer-Lemeshow test was not significant (p = 0.25), indicating the goodness of fit of the model. The Cox and Snell R and Nagelkerke R^2^ were found to be 0.211 and 0.305, respectively, indicating that the model could explain the 30.5% variability in the dependent variable for POGB being predicted accurately.

It was observed that participants with less than four family members [AOR: 0.515, CI (0.266-0.840)], duration of playing games up to three hours per day [AOR: 6.382, CI (3.363-12.111)], and using only online gaming mode [AOR: 0.539, CI (0.316-0.920)] were associated with higher odds of developing POGB, with a statistically significant p-value of <0.05.

## Discussion

The present study aimed to estimate the prevalence of POGB among professional college students and assess their psychological correlates. This study is one of the first analyses to focus on a domain that is often neglected. There is relatively little evidence-based literature available on this behavioral addiction, especially in small towns or remote hilly areas where people are thought to be less tech-savvy. The study was conducted in the sub-Himalayan region, the city of Rishikesh, which is in the process of becoming an educational hub in the state of Uttarakhand.

The overall prevalence of POGB was found to be 27.4% among students at professional colleges in Rishikesh. This finding was comparatively higher than the findings of a study conducted in 2019 among undergraduate medical students in Goa, which reported a prevalence of 8% [10]. Another study conducted in 2018 among college students in Peshawar, Pakistan reported a much lower prevalence of 1.5% for internet gaming disorder [16]. Moreover, another study [17] reported a prevalence of 17.7% among participants of 13-40 years in age. According to a narrative review [18], the prevalence of internet gaming disorder varies worldwide and is estimated to be between 0.2% and 8.5%. The difference in the prevalence of POGB might be attributed to the contextual differences in national and international settings, as well as variations in sample characteristics and ease of access to the internet. The present study also found that out of 27.4% of the participants with POGB, females accounted for the higher proportion (60.5% as compared to 39.5% for males). This finding contrasts with that of another study [10] where it was found that male participants (12.1%) were affected more by problematic online gaming compared to females (4.9%). Other studies [12,19] have also suggested a higher inclination of problematic online gaming among males than among females. Another study [20] has concluded that among the young Spanish population, males indulged in gaming for longer hours and fulfilled the criteria for heavy gaming at a much higher proportion than females (35% and 6%, respectively), which contrast with our findings. The difference may be due to the variability in the samples, as the majority of the participants in the present study were females.

The present study also found that the study participants from dental/paramedical sciences had 1.021 higher odds of developing POGB when compared to other streams such as technology, management, commerce, etc. This finding contrasts with a previous study [10], which concluded that due to busy schedules and heavy academic burden, students from medical and related fields were less exposed to frequent gaming.

Another study has revealed a significant association between the duration of gaming and POGB, which is an expected finding, as those who develop POGB tend to play these games more frequently [21]. Although there is no consensus regarding the definition of excessive gaming time, experts suggest that looking at gaming time per day is more effective than analyzing weekly time spent on gaming to assess problematic behavior. A study conducted in 2018 revealed that the average duration of gaming was three hours among the study participants [5]. In the present study, 60.5% of the participants who met the criteria for POGB played more than three hours and had 10 times higher odds of developing problematic online gaming when compared to the participants who played less than three hours. This finding is higher than that reported in another study [22], which revealed that 32.4% of adults played daily for one to two hours.

The finding of a higher preference for mobile phones/tablets for gaming in the present study (81.5%) is in line with a study conducted in 2018 among students at a medical college, which suggested that mobile phones were used by about 63% of students to access the internet and to play online games [11]. The present study found a significant association between the mode of gaming and POGB. Of the total participants, 29.8% preferred only online gaming mode, while the majority (70.2%) preferred both online and offline modes. The odds of developing POGB among participants using only the online mode for gaming was 0.3 times lower than that of participants who preferred both.

Of the 27.4% participants with POGB, 76.6% were found to be playing on a multi-player gaming format, and only 23.4% preferred the single-player format. These results are similar to those of a study that suggested that 27.5% of participants who met the addiction criteria preferred massively multi-player online role-playing games (MMORPGs) [16]. However, as per the present study, the odds of developing POGB were 0.33 times lower with multi-player gaming format, which is in contrast with the study that suggests a significant association between multi-player gaming format, especially MMORPG, and gaming addiction [23].

The association of POGB with mental well-being and depressive symptoms was found to be statistically significant in the present study. Participants with poor well-being had 3.102 times higher odds of having POGB than those with normal well-being, and participants who had issues indicative of depression had 4.375 times higher odds of having POGB. Our findings in this respect are in line with those of a study that suggested that lower well-being scores were reported by adolescents with POGB [10]. Another study has reported similar findings among adolescents in Taiwan [18]. The findings of the present study are also in line with those of Griffiths et al. [24] and Triberti et al. [25]. In addition, reasons for playing video games were differentially related to psychological functioning, with the most pronounced findings being escape-oriented, in contrast to gain-oriented motives.

Participants with mild symptoms of depression had 3.191 times higher odds of having POGB as compared to participants with no or minimal symptoms, and participants who were found to have moderate and moderately severe symptoms of depression had 2.901 times higher odds of having POGB. Fest et al. studied the implications of problematic gaming on the psychosocial well-being of respondents. The findings of the study revealed a negative association between problematic gaming and satisfaction with life. Another study has suggested that problematic online gaming often led to depressive symptoms [26]. The findings of the study conducted by Aguado et al. [20] agree with those of the present study.

It is important to be alert to and recognize this gaming pattern among adolescents and young people, which may seem alarming for parents and psychologists, and to pay attention to the intrinsic and extrinsic factors related to this behavior, which can be negative. If the behavior is not conditioned and the inclination toward the virtual world continues to increase, it will affect the overall health and well-being of the individual. POGB is one such addiction and is significantly associated with the psychological health of the individual. Therefore, despite being strict, the environment needs to be resilient and welcoming for students of professional colleges, both at educational institutes and at home.

This study has revealed few significant associations, albeit with a lack of evidence from the literature; moreover, since POGB and internet gaming disorder are relatively new phenomena, more research and studies are required to gain deeper insights into the subject.

Limitations of the study

The study was conducted during the COVID-19 pandemic, and it affected the data collection, which had to be conducted online via Google Forms. As the colleges had switched to the online mode of instruction, students mostly stayed at home. This might have had an influence on their use of screen time, and hence their gaming behavior. The cross-sectional study design is susceptible to recall bias, and the self-administered questionnaire included in the study might have led to a social desirability bias in the information provided by the participants.

## Conclusions

POGB is one of the new behavioral addictions and it is often neglected. Internet gaming disorder has recently been added to the ICD-11, although it was mentioned as a condition earlier in the Diagnostic and Statistical Manual of Mental Disorders, 5th Edition (DSM-5). The addition of this condition to ICD-11 has attracted the attention of many researchers worldwide. This study found the prevalence of POGB among professional college students in Rishikesh to be comparatively higher than the findings of other such studies in the Indian setting. There was a significant association between POGB and mental well-being, as well as depressive symptoms. More evidence and research findings are needed to bring the domain of behavioral addiction into the mainstream so that this issue can be addressed clinically.

## References

[1] (2014). Behavioral Addictions: Criteria, Evidence, and Treatment. Elsevier.

[2] Levitis DA, Lidicker WZ, Freund G (2009). Behavioural biologists don't agree on what constitutes behaviour. Anim Behav.

[3] de Castro V, Fong T, Rosenthal RJ, Tavares H (2007). A comparison of craving and emotional states between pathological gamblers and alcoholics. Addict Behav.

[4] Holden C (2001). 'Behavioral' addictions: do they exist?. Science.

[5] Yarasani P, Sultana Shaik R, Raju Myla AR (2018). Prevalence of addiction to online video games: gaming disorder among medical students. Int J Community Med Public Health.

[6] (2022). World Health Organization: addictive behaviors: gaming disorder. https://www.who.int/news-room/q-a-detail/addictive-behaviours-gaming-disorder.

[7] (2022). PWC: Confederation of Indian industry - how mHealth can revolutionise the Indian healthcare industry. PWC.

[8] Caplan SE (2007). Relations among loneliness, social anxiety, and problematic internet use. Cyberpsychol Behav.

[9] Männikkö N, Billieux J, Kääriäinen M (2015). Problematic digital gaming behavior and its relation to the psychological, social and physical health of Finnish adolescents and young adults. J Behav Addict.

[10] Bicholkar AU, Dias A, Mascarenhas V (2019). Prevalence of problematic online gaming among undergraduate medical students and its relation to well-being, self-esteem and depressive mood in Goa, India. Int J Community Med Public Health.

[11] Raju Srijampana VVG, Endreddy AR, Prabhath K, Rajana B (2014). Prevalence and patterns of internet addiction among medical students. Med J DY Patil Univ.

[12] Wartberg L, Kriston L, Thomasius R (2017). The prevalence and psychosocial correlates of internet gaming disorder. Dtsch Arztebl Int.

[13] Pápay O, Urbán R, Griffiths MD (2013). Psychometric properties of the Problematic Online Gaming Questionnaire Short-Form and prevalence of problematic online gaming in a national sample of adolescents. Cyberpsychol Behav Soc Netw.

[14] Topp CW, Østergaard SD, Søndergaard S, Bech P (2015). The WHO-5 Well-Being Index: a systematic review of the literature. Psychother Psychosom.

[15] Kroenke K, Spitzer RL, Williams JB (2001). The PHQ-9: validity of a brief depression severity measure. J Gen Intern Med.

[16] Salam Z, Sadiq Z, Tajamul U, Sethi MR, Irfan M (2019). Internet gaming disorder in students of Peshawar: a cross sectional survey. J Ayub Med Coll Abbottabad.

[17] Subramaniam M, Chua BY, Abdin E (2016). Prevalence and correlates of internet gaming problem among internet users: results from an internet survey. Ann Acad Med Singap.

[18] Naskar S, Victor R, Nath K, Sengupta C (2016). “One level more:” a narrative review on internet gaming disorder. Ind Psychiatry J.

[19] Ko CH, Yen JY, Chen CC, Chen SH, Yen CF (2005). Gender differences and related factors affecting online gaming addiction among Taiwanese adolescents. J Nerv Ment Dis.

[20] Buiza-Aguado C, Alonso-Canovas A, Conde-Mateos C, Buiza-Navarrete JJ, Gentile D (2018). Problematic video gaming in a young Spanish population: association with psychosocial health. Cyberpsychol Behav Soc Netw.

[21] Van Rooij AJ, Schoenmakers TM, Vermulst AA, Van den Eijnden RJ, Van de Mheen D (2011). Online video game addiction: identification of addicted adolescent gamers. Addiction.

[22] Choi D, Kim J (2004). Why people continue to play online games: in search of critical design factors to increase customer loyalty to online contents. Cyberpsychol Behav.

[23] Lemmens JS, Valkenburg PM, Peter J (2010). The effects of pathological gaming on aggressive behavior. J Youth Adolesc.

[24] Griffiths MD, Kuss DJ, Lopez-Fernandez O, Pontes HM (2017). Problematic gaming exists and is an example of disordered gaming. J Behav Addict.

[25] Triberti S, Milani L, Villani D, Grumi S, Peracchia S, Curcio G, Riva G (2018). What matters is when you play: investigating the relationship between online video games addiction and time spent playing over specific day phases. Addict Behav Rep.

[26] Gentile DA, Choo H, Liau A, Sim T, Li D, Fung D, Khoo A (2011). Pathological video game use among youths: a two-year longitudinal study. Pediatrics.

